# Neurobiology of cancer: Definition, historical overview, and clinical implications

**DOI:** 10.1002/cam4.4488

**Published:** 2021-12-24

**Authors:** Boris Mravec

**Affiliations:** ^1^ Institute of Physiology, Faculty of Medicine Comenius University in Bratislava Bratislava Slovakia; ^2^ Biomedical Research Center, Institute of Experimental Endocrinology Slovak Academy of Sciences Bratislava Slovakia

**Keywords:** adrenergic signaling, epinephrine, hypothalamic‐pituitary‐adrenocortical axis, innervation, neurobiology of cancer, norepinephrine, propranolol, psychoneuroimmunology, stress, sympathoadrenal system, β‐blockers

## Abstract

Studies published in the last two decades have clearly demonstrated that the nervous system plays a significant role in carcinogenesis, the progression of cancer, and the development of metastases. These studies, combining oncological and neuroscientific approaches, created the basis for the emergence of a new field in oncology research, the so‐called “neurobiology of cancer.” The concept of the neurobiology of cancer is based on several facts: (a) psychosocial factors influence the incidence and progression of cancer diseases; (b) the nervous system affects DNA mutations and oncogene‐related signaling; (c) the nervous system modulates tumor‐related immune responses; (d) tumor tissues are innervated; (e) neurotransmitters released from nerves innervating tumor tissues affect tumor growth and metastasis; (f) alterations or modulation of nervous system activity affects the incidence and progression of cancers; (g) tumor tissue affects the nervous system. The aim of this review is to characterize the pillars that create the basis of cancer neurobiology, to describe recent research advances of the nervous system's role in cancer diseases, and to depict potential clinical implications for oncology.

## Graphical abstract

Studies published in the last two decade have clearly demonstrated that the nervous system plays a significant role in carcinogenesis, the progression of cancer, and the development of metastases. These studies, combining oncological and neuroscientific approaches, created the basis for the emergence of a new field in oncology research, the so‐called “neurobiology of cancer.” The neurobiology of cancer is uncovering new pathways and mechanisms participating in tumorigenesis, proliferation of cancer cells, and the development of metastasis and created a basis for repurposing of approved drugs as well as the introduction of new drugs that might be utilized for more efficient cancer treatment and prevention.

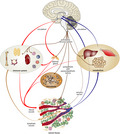

## 1 INTRODUCTION

**FIGURE 1:**
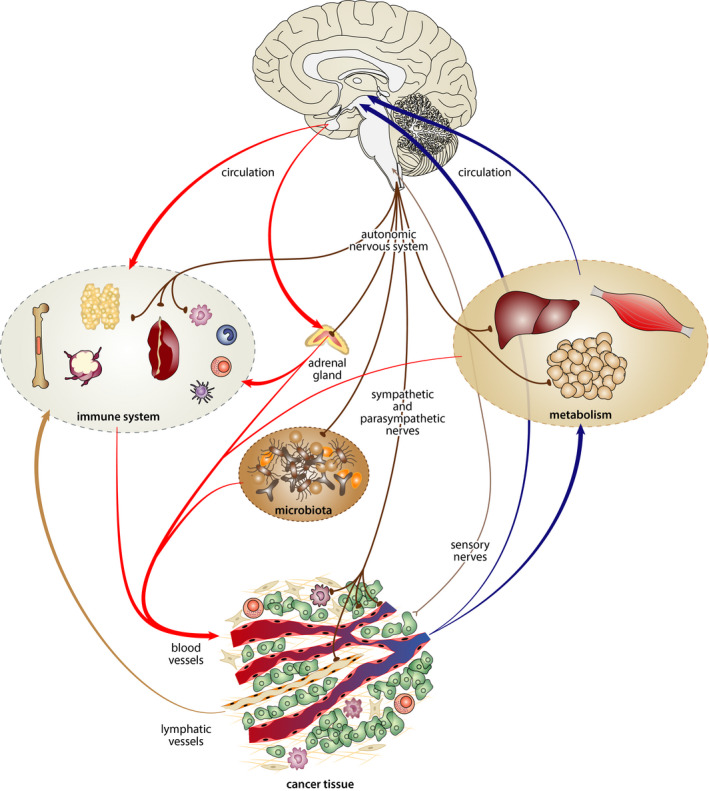
Schematic depiction of the main pathways mediating bidirectional interactions between the nervous system and tumor. The nervous system can affect a tumor: directly, through sympathetic, parasympathetic, and sensory nerves that innervate various targets within the cancer tissue (e.g., cancer cells, immune and other stromal cells, blood, and lymphatic vessels) and indirectly by modulating the activity of the endocrine glands (e.g., adrenals) and immune organs, as well as by modulation of microbiota. The tumor can affect brain activity directly via soluble mediators released from cells within the tumor microenvironment and indirectly by altering metabolism. These metabolic effects of tumors are related also to the alteration of hypothalamic functions (e.g., hypothalamic inflammation) and may contribute to dysregulation of energy balance and the development of cancer cachexia

For decades, oncological research was focused mainly on the genetic and immune factors related to cancer. This research led to the identification of tumor suppressor genes, proto‐oncogenes, myeloid‐derived suppressor cells, and other factors within the tumor micro‐ and macroenvironments that play an important role during carcinogenesis and cancer growth. However, evidences accumulated in the last 20 years have clearly shown that the processes associated with cancer initiation and progression are also significantly affected by the nervous system (Figure 1).^1^

The role of the nervous system in the modulation of physiological processes and its role in disease development and progression are the center of interest for various neurobiological disciplines, combining neuroscientific approaches with classical disciplines of medicine. Neurobiological research of diseases is traditionally focused on neurological and psychiatric disorders. For example, the *neurobiology of depression* is focused on investigation of functional and morphological alterations of the brain that participate in the development of depression, the *neurobiology of Parkinson's disease* is focused on elucidation of processes related to the loss of dopaminergic neurons in the substantia nigra. However, in recent years, researchers have also started to study the neurobiological aspects of somatic diseases. For example, the *neurobiology of obesity* is focused on investigating the role of the brain and autonomic nerves in the development of metabolic alterations leading to obesity, the *neurobiology of diabetes* is focused on elucidating the role of hypothalamic control of energy and glucose homeostasis, and the *neurobiology of hypertension* is focused on investigating the neural mechanisms participating in the maintenance of high blood pressure. Analogously, research into the modulatory effect of the nervous system on tumor initiation, progression, and the formation of metastases might be referred to as the *neurobiology of cancer*. In this sense, the term neurobiology of cancer is related to “somatic,” non‐nervous system cancers (e.g., mammary, pancreatic, ovarian, and colon cancer, in addition to hematologic malignancies) as well as cancers of the nervous system (e.g., glioblastoma). However, the term neurobiology of cancer used in this article is applied only to somatic cancers.

**FIGURE 2:**
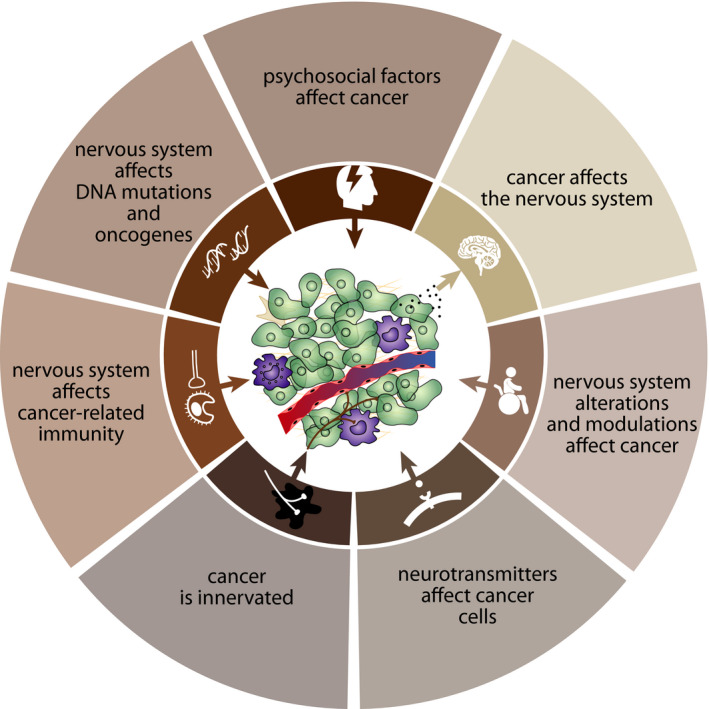
Schematic depiction of the pillars creating a basis for the neurobiology of cancer. These pillars are based on accumulated facts demonstrating that there are bidirectional interactions between the nervous system and cancer. It has been shown that psychosocial factors affect cancer incidence and progression. These effects are mediated, at least partially, by neurotransmitters released by nerves innervating cancer tissue. Neurotransmitters released by autonomic nerves affects DNA mutations and oncogene‐related pathways, stimulates cancer cell proliferation, and modulates cancer‐related immunity. The role of the nervous system in cancer is further documented by findings that alterations or modulation of nervous system activity significantly affects cancer incidence and progression. Conversely, cancer also affects brain functions, which might participate in the development of cancer cachexia, for example

The concept of the neurobiology of cancer stems from interdisciplinary research situated at the borderline of oncology and neurosciences. This concept is based on several pillars: (a) psychosocial factors influence the incidence and progression of cancer diseases; (b) the nervous system affects DNA mutations and oncogene‐related signaling; (c) the nervous system modulates tumor‐related immune responses; (d) tumor tissues are innervated; (e) neurotransmitters released from nerves innervating tumor tissues affect tumor growth and metastasis; (f) alterations or modulation of nervous system activity affects the incidence and progression of cancers; (g) tumor tissue affects the nervous system (Figure 2).

The aim of this article is to provide a historical overview of the findings on which the concept of neurobiology of cancer is based. These findings will not only contribute to a better understanding of the complexity of the etiopathogenesis of cancer, but also create the basis for new therapeutic and preventive approaches in oncology.

### 1.1 Psychosocial factors affect tumor incidence and growth

For centuries, physicians observed and discussed the role of psychosocial factors in the development and progression of various diseases, including cancer. Based on the approaches they used, three periods related to the role of psychosocial factors in cancer diseases can be recognized, reflecting the prevailing scientific methods used during a given period.

#### 1.1.1 Period of empirical evidences (melancholy period)

Scientific and literary texts mentioning the relationship between psychosocial factors, especially melancholy mood, and cancer initiation and progression have accumulated from antiquity.^2^ The first preserved text describing the possible influence of psychological factors on the incidence of tumors can be traced back to the second century AD in the treatise *De Tumoribus* of Galen of Pergamon. Galen, who espoused the Hippocratic tradition that cancer is associated with an excess of black bile, believed that melancholy represents a factor responsible for cancer.^3^ This idea dominated medicine for a long time. For example, the Byzantine physician Aetius hypothesized that tumors are the result of “melancholy accumulating in the brain.” Centuries later, Arabic physicians such as Avicena and Avenzoar similarly reported that the development of tumors was associated with a melancholy mood.^2^ In the following centuries, a large number of physicians continued to believe that a melancholy mood formed the basis for the development of tumors. For example, at the beginning of the 17^th^ century, the French surgeon Claude Chapuys de Saint‐Amour noted in his *Treatise on cancer*, *as occult as ulcerated* that tumors are caused by grief, anger, and agitation.^4^ Similarly, Guillaume de Houppeville mentioned that sadness, compassion, grief, and excessive workload can exacerbate melancholy and cancer development.^5^ In 1740, the French surgeon Gilles Le Vacher noted in his work on breast cancer that significant and persistent grief could result in the development of breast cancer.^6^ Based on the above considerations, cancer and melancholy began to become synonymous, and people began to perceive cancer as a consequence of grief.^2^ In the early 18^th^ century, the French physician Claude Deshais Gendron pointed to a link between serious life situations and the incidence of cancer.^7^ Several years later, English physicians such as J.A. Burrows, T.W. Nunn, and R. Stern described the role of psychological factors, including hypersensitivity and frustration, to the incidence of tumors in women.^2^, ^8^ In 1802, the French physician J.B.A. Burdel stated in essay *Le cancer des mamelles*, that women prone to breast cancer have a specific psychological profile. He hypothesized that the main cause of tumors in these women was suffering due to aging and the loss of beauty. He further noted that adverse life events, such as the dangerous situations in which women found themselves during the French Revolution, contributed to the increased incidence of breast cancer, especially in nuns.^9^ Similar considerations were made in 1807 by Viel Haut Mesnil, who hypothesized that aging and depression could affect the genitals and increase the likelihood of tumors in these organs.^10^ Description of the role of psychosocial factors in cancer was also published in the mid‐19^th^ century by Walter H. Walshe in *The Nature and Treatment of Cancer*.^11^ In 1870, an English surgeon, Sir James Paget in his classic Surgical Pathology mentioned that emotional disturbances, such as deep anxiety, deferred hope, and disappointment are quickly followed by the growth and increase of cancer.^12^ Fifteen years later the United States surgeon Willard Parker published a book in which he suggested that great mental depression, particularly grief, induces a predisposition to such diseases as cancer, or becomes an precipitating cause under circumstances where the predisposition had already been acquired.^13^

#### 1.1.2 Period of statistical and epidemiological studies

In the last decades of the 19^th^ century, advances in statistics and epidemiology made it possible to examine more precisely the relationship between psychosocial factors and cancer. In 1893, the London surgeon Herbert Snow conducted an epidemiological study involving 250 patients. He found that of the 250 patients diagnosed with breast or uterine tumors, 43 had a history of suspected mechanical injury, with 15 of these 43 patients reporting recent problems. Thirty‐two other patients said they had jobs involving hard work and were in need. Moreover, 156 patients were identified as having recent, serious life problems, such as the loss of a loved one. Only 19 patients did not show any of the above factors.^14^ In 1926, psychologist Elida Evans published a book in which 100 patients with cancer had all lost or had significant emotional bonds disrupted before they developed cancer.^2^, ^8^, ^15^

#### 1.1.3 Period of psychoneuroimmunological studies

Elucidation of the regulatory effects of the nervous system on the endocrine^16^, ^17^ and immune systems^18^ led to the establishment of a new research area, psychoneuroimmunology.^19^ The psychoneuroimmunological view of somatic diseases provided the basis for studies investigating the mechanisms and pathways interconnecting psychosocial factors and cancer. One of the first studies was performed by Spiegel et al. in 1989. These authors investigated the effect of psychotherapy on cancer survival and showed that group psychotherapy aimed at reducing anxiety, depression, and pain in women with metastatic breast cancer also prolonged survival.^20^ Later, Fawzy, et al.^21^ showed that a 6‐week structured group psychological intervention designed to improve stress management effectiveness reduced recurrence and prolonged survival in patients with melanoma. However, studies published later in 2001 and 2007, as well as two meta‐analyses published in 2004, did not find that psychotherapy had an effect on the survival of patients with breast cancer.^22^, ^23^, ^24^, ^25^, ^26^ In the following years, a large number of studies were conducted and faithful meta‐analyses were published, some of which showed a positive effect of psychotherapy on the survival of cancer patients, while others did not confirm this effect. Analysis of these studies suggests that there are several factors, such as the type of intervention (group vs. individual therapy), intensity, frequency, and duration of treatment that determine their effectiveness.^27^ In addition, as suggested by Mirosevic et al.,^28^ psychotherapy may preferentially prolong survival in specific subgroups of patients, especially in socially isolated cancer patients. Due to this social isolation, these cancer patients may have a higher level of hopelessness and despair. It is known that these factors adversely affect the onset and progression of cancer, as evidenced by a series of earlier clinical studies conducted by Schmale and Iker. In one of the first studies, published in 1966, Schmale and Iker found that the presence or absence of cervical cancer could be determined in asymptomatic patients with cytologically confirmed dysplasia through an interview to determine the potential for hopelessness/despair, or the recent experience of these feelings. Based on this interview, they were able to correctly identify 8 of 14 women with cancer and 23 of the 26 healthy women.^29^

Whereas the first description of the role of psychosocial factors in cancer is dated almost 2 millennia ago, the mechanisms and pathways interconnecting melancholy mood, adverse life events/stress, and depression with cancer incidence and progression only started to be elucidated in more detail by researchers utilizing approaches that included neuroscientific methods since the beginning of 21^st^ century. This research has provided a mechanistic explanation of how psychosocial factors might affect cancer.^30^

### 1.2 The nervous system affects DNA mutations and oncogene‐related signaling

Alterations at the level of DNA play a crucial role in cancer initiation, progression, and metastasis. Several factors, including radiation, oncogenic viruses, and chemical carcinogens, might induce changes at the level of DNA that lead to the transformation of normal cells to cancer cells and subsequent cancer progression. Recently, it was found that the nervous system also participates in the development of DNA mutations. In addition, the nervous system attenuates DNA repair and sensitizes the cell to mutagenic factors. These data indicate that the nervous system plays a significant role in the first step responsible for initiating cancer, as well as processes related to cancer growth and metastasis. The “pro‐mutagenic” effects of the nervous system are mediated mainly via systems responsible for the neuroendocrine stress response, particularly the sympathoadrenal system and hypothalamic‐pituitary‐adrenocortical axis.

Recently, molecular mechanisms responsible for the pro‐mutagenic potential of the effector molecules released by neuroendocrine stress response systems were elucidated. It was shown that epinephrine, norepinephrine, and cortisol might induce alterations in target cells at the level of DNA via several mechanisms, including induction of DNA mutations, suppression of DNA repair, and by activation of oncogene‐related intracellular pathways.

#### 1.2.1 Nervous system effects on DNA mutations

As early as in 1925, Cramer^31^ published the first study investigating the role of the nervous system in chemical carcinogenesis. However, it was only at the beginning of the 21^st^ century that the mechanisms responsible for modulating the effects of the nervous system on DNA mutations started to be elucidated at the cellular and molecular levels. It was found that the nervous system might affect DNA mutations via at least three mechanisms, including potentiation of mutagenesis, reduction of DNA repair, and sensitization of cells to mutagens.

In 2007, Flint et al.,^32^ measured the effect of stress hormones and neurotransmitters such as epinephrine, norepinephrine, and cortisol on DNA using in vitro murine 3T3 cells. The authors demonstrated that short‐term exposure (<30 min) to physiological concentrations of these molecules significantly increased DNA damage in 3T3 cells. Moreover, cortisol and norepinephrine also interfered with the repair of DNA damage in cells exposed to UV radiation. A targeted gene array showed that cortisol, norepinephrine, and epinephrine affected the transcription of several genes, including the proto‐oncogene CDC25A. A few years later, Hara, et al.^33^ showed that the sympathoadrenal system also plays a role in regulating the functions of tumor suppressor genes. The authors observed that activation of β‐adrenergic receptors initiated a signaling cascade that induced the degradation of p53, the product of a tumor suppressor gene in mice and human cell lines. In 2012, Feng et al.^34^ demonstrated that chronic restrain stress greatly promoted ionizing radiation‐induced tumorigenesis in p53(+/−) mice. This effect of stress was mediated by glucocorticoids, which increased mouse double minute 2 homolog (MDM2) activity and decreased p53 function. Later, Hara et al.^35^ showed that stress‐induced DNA damage mediated by β‐adrenergic signaling is preventable by an antagonist of β‐adrenergic receptors. Two year later, Reeder et al.^36^ demonstrated that the stress hormones norepinephrine and cortisol‐induced DNA damage in triple‐negative breast cancer cells.

#### 1.2.2 Nervous system's effects on oncogene‐related signaling

It has been shown that nervous system‐related signaling is also connected to the function of proto‐oncogenes and tumor suppressor genes. The role of the sympathoadrenal system in activating proto‐oncogenes was demonstrated in 1988, by Kousvelari et al.,^37^ who showed that stimulation of β‐adrenergic receptors by isoproterenol induced expression of the proto‐oncogene c‐fos in rat parotid acinar cells in vitro. A few years later, Iwaki et al.^38^ and Okazaki et al.^39^ showed that activation of α‐ and β‐adrenergic receptors induced the expression of c‐fos and c‐jun proto‐oncogenes in rat arterial smooth muscle and myocardium. However, it is necessary to note that even if c‐fos and c‐jun are implicated in carcinogenesis for some cancers,^40^ they do not play a prominent role in human carcinogenesis.

Later, in vitro studies showed that the sympathoadrenal system also plays a role in the activation of proto‐oncogenes implicated in human cancers. For example, in 2011, Shi et al.^41^ demonstrated that catecholamines stimulate Her2 mRNA expression and promoter activity in human breast cancer cells via β_2_‐adrenergic receptors. Later, in 2013 Armaiz‐Pena et al.^42^ showed that activation of β‐adrenergic receptors activated Src‐related phosphoproteomic signaling networks in human ovarian cancer cells.

### 1.3 The nervous system affects tumor‐related immune response

The fact that the initiation and progression of cancer are closely and comprehensively related to the activity of the immune system has been documented by in vitro studies, in vivo experiments on animal models of cancer, as well as clinical studies. These studies have shown that the immune system recognizes cancer cells and is able to eliminate them, but it is also able to potentiate cancer initiation, progression, and the development of metastases. Research on the interactions between cancer and the immune system has shown that essentially all innate and acquired immune effector mechanisms are involved in the recognition of cancer cells and the modulation of cancer growth.^43^ The importance of the immune system's role in cancer documents the Nobel Prize in Physiology or Medicine in 2018, that received James P. Allison and Tasuku Honjo for their revolutionary advancement in cancer therapy based on immune checkpoint inhibitors.^44^

In last two decades a large number of papers have demonstrated that the nervous system modulates cancer‐related immune system activity. Recently, several mechanisms and pathways that enable the nervous system to modulate cancer initiation and progression have been described in detail resulting in the emergence of potential therapeutic implications. For example, the nervous system might modulate cancer initiation/promoting chronic inflammation, potentiate or suppress anti‐cancer immunity, as well as inhibit or stimulate the activity of cancer growth‐promoting immune cells. Therefore, the role of the nervous system in modulating immune responses to cancer is at the center of research on the neurobiology of cancer.

#### 1.3.1 Immune system plays crucial role in cancer

One of the first description of the role of immune system in cancer provided Rudolf Virchow. In 1863, he mentioned a relationship between inflammation and cancer based on his observation of inflammatory infiltrates in solid cancers.^45^ About 50 years later, Paul Ehrlich formulated the hypothesis that the host defense may prevent neoplastic cells from developing into cancers.^46^ However, this hypothesis was not proven experimentally as at this time experimental tools and knowledge were inadequate. Few years later, Murphy and Morton^47^ demonstrated significant increase of circulating lymphocytes in mice inoculated by cancer cells.

Later experiments have shown that in immunodeficient mice is increased incidence of tumors and that these animals are more susceptible to transplanted of chemical carcinogen‐induced cancers. In addition, it was observed that immunosuppressed patients have increased incidence of some cancers. Based on these findings, Frank Mac Farlane Burnet suggested that immune system react to cancer cells neo‐antigens. He formulated the immune surveillance theory stating that when small accumulation of cancer cells develop, the neo‐antigens that they possess provoke an effective immunological reaction that lead to regression of the cancer and no clinical hint of its existence.^48^ This hypothesis stated that sentinel thymus‐dependent cells of the body constantly surveyed host tissues for nascently transformed cells.^49^

However, as knowledge about the role of the immune system in cancer grew, it started to be recognized that immunosurveillance represents only one dimension of the complex relationship between the immune system and cancer. Later, Dunn et al.^50^ proposed a new hypothesis of cancer immunoediting. This hypothesis states that the immune system may also promote the emergence of primary tumors with reduced immunogenicity that are capable of escaping immune recognition and destruction. Cancer immunoediting represents a dynamic process that includes three phases: elimination, equilibrium, and escape. Whereas elimination represents the classical concept of cancer immunosurveillance, equilibrium is the period of immune‐mediated latency after incomplete tumor destruction in the elimination phase, and escape refers to the final outgrowth of cancers that have broken the immunological restraints of the equilibrium phase.^51^

#### 1.3.2 Stress and immunity

One of the first demonstrations of the modulatory effect of psychosocial factors on the immune system was provided in 1936 by Hans Selye, who described the effect of chronic stress on the thymus, spleen, and other immune organs.^52^ In subsequent mechanistic studies of neuroendocrine stress response, he showed that this effect is mediated by the HPA axis, specifically by glucocorticoids released from adrenals. In 1950, Slocumb et al.^53^ showed that glucocorticoids exert potent immunosuppressive effects in patients with inflammatory disease. Later, it was demonstrated that immune functions are also modulated by prolactin and growth hormone released from the anterior pituitary gland.^54^ These findings have shown that the brain might regulate the activity of immune cells via modulation of endocrine gland secretion.

#### 1.3.3 Psychoneuroimmunology

A new view on the mechanisms interconnecting the brain and immune function was provided by an experiment published in 1975 by Robert Ader and Nicholas Cohen, who by using Pavlovian conditioning, demonstrated that immune responses might also be conditioned.^18^ Subsequent experiments have shown that autonomic nerves innervate all immune organs and modulate the growth and release of immune cells from bone marrow^55^ Mechanistic studies have demonstrated that whereas the sympathoadrenal system modulates immune function via norepinephrine released from sympathetic nerves, as well as epinephrine and norepinephrine released from adrenal medulla, the parasympathetic nervous system modulates immunity via acetylcholine.^56^ Moreover, it was demonstrated that sensory nerves can regulate local immune responses, for example in the skin.^57^

In addition to the direct modulatory effects of signaling molecules of the nervous and neuroendocrine system on immune cells, the nervous system can also affect immune functions indirectly through modulating the growth and maturation of immune cells within the bone marrow, as well as their release to systemic circulation. This is done by influencing blood flow though immune organs and vasomotor reactions in the gastrointestinal tract, as well as through the retino‐hypothalamic tract and subsequent regulation of circadian rhythms, or via regulation of food intake and nutritional status of the organism.^58^

There are many factors that determine the effect of the nervous system on immune functions, including the type of neurotransmitter or hormone, subtype of corresponding receptors, and intensity and duration of receptor activation. For example, whereas the acute effect of psychosocial factors (stressors) on immunity seems to be predominantly stimulating, chronic exposure to adverse psychosocial factors suppresses, or dysregulates immune responses.^59^

Importantly, interactions between the nervous and immune systems are bidirectional. On one side, immune functions can be modulated by hormones released from neuroendocrine systems, as well as by neurotransmitters and neuromodulators released from nerve endings. On the other side, the immune system is able to affect both the peripheral and central nervous system via cytokines and other molecules synthetized by immune cells.^60^, ^61^

#### 1.3.4 Neuroimmunology of cancer

Psychoneuroimmunological studies have shown that the nervous system exerts a complex effect on immune functions, including anti‐cancer immunity. Therefore, in the last decade neuroimmunological research has also focused on investigating the effect of stressors and other factors affecting nervous system activity on immune parameters related to cancer initiation and progression. This research has uncovered mechanisms and pathways that interconnect the nervous system and cancer indirectly, via the immune system. It was shown that the nervous system, via humoral and nervous pathways, affect several components of the immune system that are related to cancer development and progression.

In 1999, Kalinichenko et al.^62^ showed that in vitro norepinephrine inhibited the generation of cytotoxic T lymphocytes via β‐adrenergic receptor signaling. Later, it was shown that the sympathoadrenal system significantly affected the activity and distribution of NK cells,^63^ as well as density and survival of myeloid‐derived suppressor cells in tumors and other tissues, along with the expression of immunosuppressive molecules by these cells.^64^

Neuroimmunological studies of cancer have provided a mechanistic explanation of how adverse psychosocial factors might affect cancer. However, the effect of the nervous system on cancer‐related immunity is highly complex, as different components of nervous system affect the activity of various subtypes of immune cells differently.

### 1.4 Tumor tissue is innervated

Peripheral nerves play an important role during the development of an organism, participate in homeostatic regulations of innervated organs and tissues, modulate repair and regeneration of damaged tissues, and participate in compensatory reactions of tissues affected by diseases.^65^ In addition, recent data have demonstrated that peripheral nerves also play an important role in the progression of various pathological processes. For example, signals transmitted by peripheral nerves participate in the development of neurogenic hypertension, obesity, and diabetes. Recent data have shown that peripheral nerves innervating cancer tissue also play an important role in the initiation and progression of cancer.^66^, ^67^

Even if the first description of cancer tissue innervation was published more than 100 years ago, factors participating in the ingrowth of new nerves into cancer and mechanisms mediating the stimulatory effect of these nerves on cancer progression and the development of metastases have only recently been elucidated.

The investigation of nerves role in cancer can be divided into two periods, further subdivided into several parts. These two waves of interest in the study of cancer innervation and its role, characterized by utilization of the available research methods during a given period (especially histological methods), were interrupted by a period when significant new discoveries related to cancer biology and therapy shifted general interest in oncology to research related to chemotherapy, cancer immunity, neovascularization, and other factors.

#### 1.4.1 Period of discovery of cancer innervation

This period, which started from the second half of the 19^th^ century and finished at the end of the first half of the 20^th^ century, can by subdivided into two parts.

#### 1.4.2 Virchow period

Even if speculation about the role of nerves in cancer development might have been already begun in the 18^th^ century, Rudolf Virchow, the founder of cellular pathology and one of the pioneers of tumor research, did not assign a more important role to nerves in the origin and growth of cancer (for review see^68^). Based on his opinion, it was assumed that tumor tissue was not innervated.^69^ However, several years later, Young published a paper demonstrating the presence of methylene‐blue‐stained nerves in breast and cervical cancer, as well as in sarcoma.^70^ In 1903, Cheatle published clinical observations indicating that a relationship exists between the spread of the primary cancer and the distribution of nerves or their trophic areas.^71^ The role of nerves in the development of cancer was also mentioned by Ewing in his textbook on tumors published in 1919.^72^

#### 1.4.3 Silver staining period

In 1928, Oertel showed the presence of nerves in cervical cancer and adenocarcinoma of the rectum in silver‐stained specimens.^73^ Three years later, he described the presence of silver‐stained nerve structures in breast cancer, uterine myoma, and fibrosarcoma.^74^ In 1949, Shapiro and Warren in a combined morphological and functional study demonstrated the presence of nerves in Brown‐Pearce carcinoma and mouse mesothelioma anterior ocular chamber transplants. In addition, they showed that sympathetic nerve stimulation induced the contraction of vessels in these tumors.^75^ Furthermore, in 1956, Winkelmann described the presence of silver‐stained nerves in squamous cell carcinoma and basal cell carcinoma of the eyelid.^76^

#### 1.4.4 Period of “rediscovery” of cancer innervation

This period, dated at the end of 20^th^ century, represents a new wave characterized by the use of modern methods enabling precise investigation of cancer innervation. This period can be subdivided into two parts.

#### 1.4.5 Electron microscopy and immunohistochemistry period

This period is characterized by more detailed descriptions of the phenotypes of nerves innervating tumor tissues. In 2001, Seifert and Spitznas demonstrated the presence of nerves in pigmented and one non‐pigmented adenoma of the ciliary body epithelium using electron microscopy.^77^ Later, Seifert et al. showed nerve‐like structures immunoreactive for protein gene product 9.5 and vasoactive intestinal neuropeptide in urinary bladder tumors.^78^ In 2007, Palm and Entschladen published the hypothesis that tumor cells may induce their own innervation, a process they have termed “neoneurogenesis” Moreover, they called the structures that enable interaction between peripheral neurons and tumor cells a “neuro‐neoplastic synapsis”^79^ Importantly, further immunohistochemical studies showed that the density of innervation and size of nerves determines, or at least correlates with the aggressiveness of the tumor.^80^, ^81^, ^82^, ^83^, ^84^

#### 1.4.6 Genetics and electrophysiology period

In 2019, Kamiya et al. employed a series of genetic techniques enabling them to selectively manipulate (stimulate) sympathetic or parasympathetic nerves innervating chemically induced breast tumors in rats.^85^ They showed that stimulation of sympathetic nerves in tumors accelerated cancer growth and progression, whereas stimulation of parasympathetic nerves had the opposite effect. Later, in 2020 McCallum et al. published a paper describing chronic neuronal activity recorded from breast tumors in mice showing that neural electrical activity is present within mammary tumors. As the authors stated, their results indicate a strong connection between the autonomic nervous system and tumors.^86^

**FIGURE 3:**
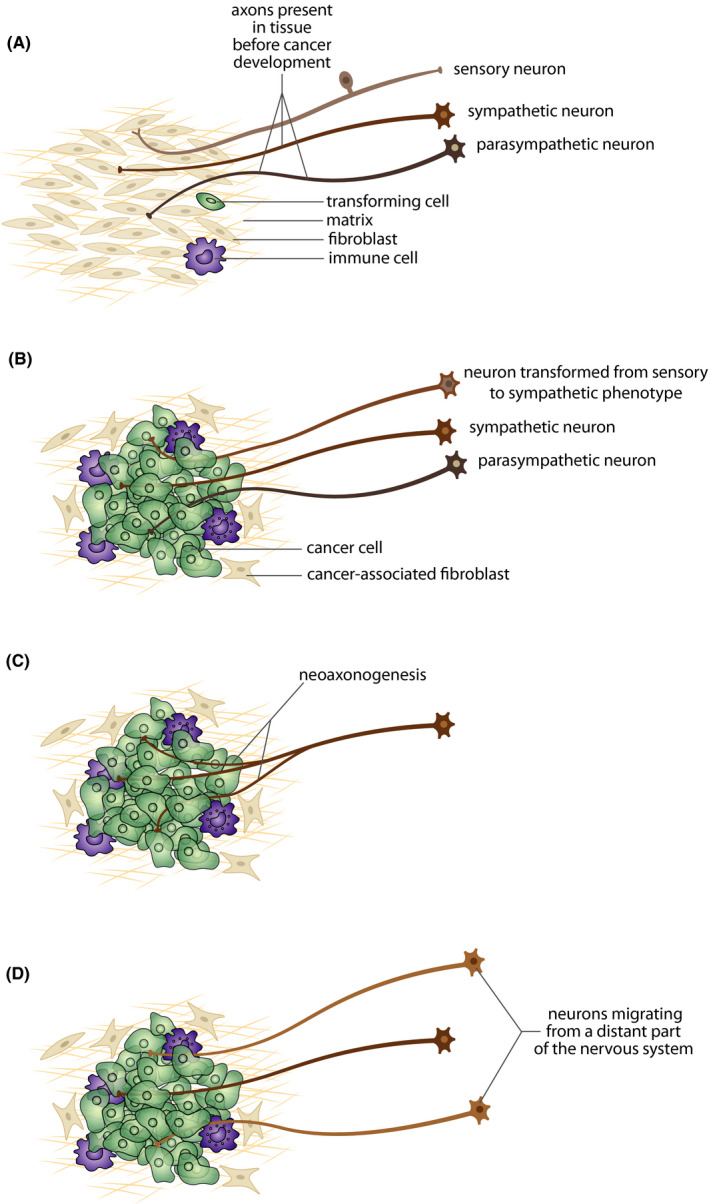
Schematic depiction of the origin of nerves innervating tumor tissue: (A) sympathetic, parasympathetic, and sensory nerves already present in tissue before the transformation of normal tissue cells into cancer; (B) sensory neurons innervating the tissue of tumor origin that have phenotypically transformed to sympathetic neurons; (C) new branches of nerves growing to the tumor tissue from nerves localized around the tumor tissue; (D) new neurons migrating from a distant part of the central or peripheral nervous system into the tumor tissue or its vicinity

Available data indicate that there are several sources of nerves innervating tumor tissue: (a) nerves already present in tissue before the transformation of normal tissue cells into cancer; (b) phenotypically transformed neurons that innervate the tissue of tumor origin^87^; (c) new branches of nerves growing to the tumor tissue from nerves localized around the tumor tissue; (d) new neurons migrating from a distant part of the central or peripheral nervous system into the tumor tissue or its vicinity^88^ (Figure 3). Published data indicate that cancer manipulates the nervous system by inducing the growth of new sympathetic nerves into the tumor tissue and by trans‐differentiation of a sensory neuronal phenotype to adrenergic in order to utilize the stimulatory effect of adrenergic signaling for promoting cancer growth and development of metastasis. Besides sympathetic^89^ and parasympathetic nerves,^90^ sensory nerves also play a role in cancer,^67^, ^91^ even if their role in cancer development and progression has been described in less detail.

Interestingly, recent data indicate that cancer cells themselves may transform to a neuron‐like phenotype characterized by development of neurite‐like protrusions. These protrusions might participate in the formation of synapses between neurons and cancer cells, further potentiating the stimulatory effect of nerves on cancer growth.^92^

**FIGURE 4:**
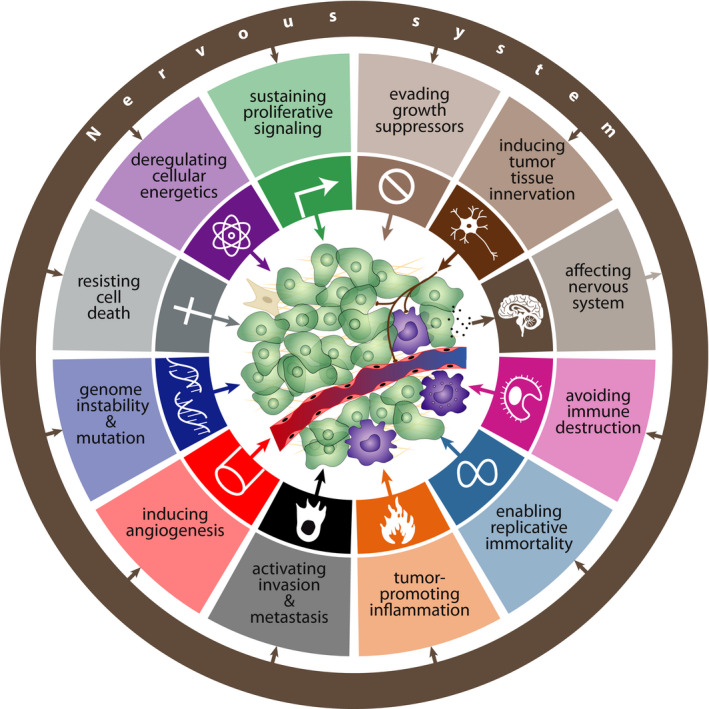
Based on the neurobiology of cancer, two additional hallmarks were added into the classical scheme of hallmarks of cancer as originally defined by Hanahan and Weinberg.^139^ These two new hallmarks include “inducing tumor tissue innervation” and “affecting nervous system” (e.g., cognitive impairment, hypothalamic inflammation‐related cancer cachexia). Importantly, all original hallmarks of cancer are under the influence of the nervous system

Accumulated evidences have clearly shown that innervation of tumor tissue represents a complex phenomenon. In support of this, tumor tissue innervation has been found to be an important factor influencing the tumor microenvironment in several cancer types. Recent studies have shown that peripheral nerves, including sympathetic, parasympathetic, and sensory nerves, interact with tumor cells and other cells of tumor tissue, and stimulate the initiation and progression of a whole spectrum of solid and hematological tumors. In addition, the tumor itself has been found to promote its own innervation, which in turn promotes tumor growth.^67^ Based on these findings, tumor innervation can be included among the basic hallmarks of cancer (Figure 4).^93^ However, more detailed mapping of the innervation of different cancers is needed. For example, it will be necessary to determine whether cancer innervation represents a general phenomenon, which types of nerves are responsible for innervation of different types of cancers, and which factors determine it.

### 1.5 Neurotransmitters affect tumor microenvironment

The innervation of cancer tissues is now an accepted fact. Autonomic and sensory nerves innervating cancer release neurotransmitters such as norepinephrine, acetylcholine, substance P, and others into the tumor microenvironment. Once there, these neurotransmitters can affect almost all hallmarks of cancer. In addition to their effect on the tumor microenvironment, neurotransmitters might also affect cancer via its effects on the tumor macroenvironment. The research of neurotransmitter effects on cancer has also focused on the effect of hormones released by the neuroendocrine system, especially epinephrine.

#### 1.5.1 Early studies investigating the presence and affinity of adrenergic receptors on cancer cells

The presence of β‐adrenergic receptors on cancer cells and their binding affinity to compounds including norepinephrine, epinephrine, and other agonists of β‐adrenergic receptors has been investigated since the 1970’s. However, this research focused on cancers of the endocrine glands and brain and therefore cannot be seen as a precursor to research on the neurobiology of cancer.

In 1989, Marchetti et al.^94^ published a study describing the presence of β‐adrenergic receptors on membranes of mammary cancer induced by a chemical carcinogen and their affinity to several compounds, including norepinephrine and epinephrine. The authors mentioned that the presence of β‐adrenergic receptors in mammary cancers might be related to catecholamines’ effect on cancer tissue. Next year, Vandewalle et al.^95^ described the presence of β‐adrenergic receptors expressed on breast cancer cells and their affinity of isoproterenol, epinephrine, and norepinephrine. However, even if the authors discussed the importance of stimulated cAMP production by these compounds, they stated that the role of these receptors in breast cancer needed more detailed study. In the following year, several studies were published focused on investigating the presence and affinity of adrenergic receptors on various types of cancers. However, these studies did not investigate the effect of adrenergic receptor activation on cancer initiation or progression.

#### 1.5.2 Studies investigating the effect of neurotransmitters on cancer incidence and progression

In 1998, Tatsuta et al.^96^ published one of the first studies investigating the effect of adrenergic receptor stimulation on cancer. The authors showed that long‐term administration of the norepinephrine‐mimicking agent metaraminol and the α_1_‐adrenergic agonist phenylephrine significantly increased the incidence of *N*‐methyl‐*N*′‐nitro‐*N*‐nitrosoguanidine‐induced gastric cancer in rats. The authors concluded that adrenoreceptor stimulation enhances gastric carcinogenesis. In 2001, Masur et al.^97^ demonstrated in vitro that norepinephrine, via β_2_‐adrenergic signaling, induced migration of SW 480 colon carcinoma cells. In the following years, it was demonstrated that norepinephrine can influence the tumor progression of some solid epithelial tumors, including nasopharyngeal carcinoma, ovarian cancer, and melanoma, as well as lymphoid tumors and multiple myeloma, by modulating the expression of pro‐angiogenic and pro‐metastatic factors, such as vascular endothelial growth factor.^98^, ^99^, ^100^, ^101^

In the last 20 years, the number of papers describing the effect of neurotransmitters on various aspects of cancer diseases has risen and a more detailed description of the mechanisms that mediate the effects of neurotransmitters on cancer has been provided. These studies have shown that besides norepinephrine, neurotransmitters such as acetylcholine, neuropeptide Y, GABA, and others might also affect carcinogenesis and cancer progression (for review see^102^) and that these effects might be significantly reduced by antagonists of neurotransmitter receptors, such as propranolol. Several trials investigating the efficacy of propranolol in the treatment of various cancers are currently underway.

### 1.6 Changes in signal transmission from the nervous system to peripheral tissue affect cancer incidence and progression

Indeed, the fact that the nervous system plays an important role in cancer is best documented by animal experiments and clinical studies investigating the effects of impaired signal transduction between the nervous system and peripheral tissues on the incidence and progression of cancer. For example, increased sympathetic nerve activity and cancer incidence has been documented in subgroups of patients with hypertension, in women with polycystic ovary syndrome, obese individuals, as well as in individuals exposed to cold, or those who smoke combustible or electronic cigarettes containing nicotine. On the other hand, several retrospective clinical studies have shown that reduced transmission of signals from the nervous system to peripheral tissues as a consequence of traumatic spinal cord injury, surgical vagotomy, or administration of β‐blockers is associated with reduced cancer incidence and slowed progression of cancer diseases. Although there are several other factors, in addition to the nervous system, related to these pathological conditions that may influence the incidence and progression of cancer, these studies best confirm the validity of the concept of neurobiology of cancer. In addition, recent prospective studies have shown that pharmacological reduction of signals transmitted from the nervous system to the tumor micro‐ and macroenvironments might significantly affect the progression of cancer disease in cancer patients.^103^, ^104^

From an historical point of view, it is possible to recognize two periods of scientific interest in the effects of altered or modulated transmission of signals from nerves to peripheral tissues on cancer incidence and progression.

#### 1.6.1 Early animal denervation studies

After the first descriptions of the presence of nerves in tumor tissues appeared in scientific literature, investigators started to speculate as to whether these nerves can potentiate or inhibit carcinogenesis. One of the first studies on the role of nerves in carcinogenesis was published in 1917 by Adler and Sittenfield, who showed that all rats inoculated by Flexner‐Jobling carcinoma into denervated testes very promptly developed exceptionally large and rapidly growing tumors, whereas control rats inoculated by tumors into testes with preserved innervation did not develop any tumors.^68^ On the contrary, in 1925 Cramer demonstrated that mice with denervated skin developed less tar painting‐induced tumors when compared to mice in which skin had preserved innervation.^31^ This is the first experimental study clearly showing that the nervous system potentiates cancer development.

#### 1.6.2 Later animal and clinical denervation, pharmacological, and genetic studies

From 1988, several retrospective studies were published documenting an increased risk of developing gastric cancer in patients who underwent vagotomy for the treatment of gastric ulcers.^105^, ^106^, ^107^, ^108^ Later, alternative explanations regarding the effect of transecting the vagus nerve branches innervating the stomach on the development of gastric cancer were provided.^109^, ^110^ However, the data from initial clinical trials stimulated research into the effect of interrupting signal transmission by nerves on cancer incidence and progression in animal models of cancers.

Experiments utilizing the chemical destruction of sympathetic or sensory nerve endings or transection of parasympathetic nerves have shown that sympathetic nerves exert a stimulatory effect on cancer development and progression,^111^, ^112^ whereas data obtained from experiments investigating the role of parasympathetic and sensory nerves were ambiguous.^113^, ^114^, ^115^ Interestingly, in 2013 Magnon et al. published a paper showing that the early phase of prostate cancer development in mice might be prevented by chemical or surgical sympathectomy, whereas cancer dissemination might be attenuated by pharmacological blockade, or genetic ablation of M_1_ muscarinic receptors.^80^ These data indicate that sympathetic and parasympathetic nerves affect different phases of prostate cancer. Later in 2014, Zhao et al. showed that surgical or pharmacological vagotomy, or administration of muscarinic M_3_ receptor antagonists reduced gastric tumorigenesis.^116^

The role of the nervous system in cancer is also supported by findings from retrospective clinical studies determining the incidence of cancer in patients with lesion of nervous system. For example, several studies have shown that there is a reduced incidence of prostate tumors in patients with traumatic spinal cord injury.^117^, ^118^, ^119^ Based on these findings, Rutledge et al.^120^ hypothesized that the processes of tumorigenesis in the prostate show a high degree of dependence on the activity of autonomic nerves innervating this organ. This hypothesis is also supported by abovementioned findings from preclinical studies that have shown an important role for both sympathetic and parasympathetic nerves in the development of prostate tumors.^80^ Another clinical example indicating the importance of the nervous system in cancer is represented by polycystic ovary syndrome (PCOS), which is characterized by increased cancer risk as well as exaggerated sympathetic nerve activity.^121^, ^122^ Importantly, it is suggested that exaggerated sympathetic nerve activity might participate in this observed increased cancer incidence in PCOS.^123^ In addition, the observed, increased cancer incidence in patients with hypertension or obesity might result, at least partially, from the increased sympathetic nerve activity documented in these groups of patients. In support of this, the interruption of signal transmission from sympathetic nerves to effector cells by β‐blockers also affects the incidence and progression of cancer in patients with hypertension.

Besides retrospective clinical studies evaluating the effects of therapeutic interventions or accidental lesions affecting peripheral nerves, investigation of the effects of targeted lesions and stimulation of brain structures in laboratory animals on cancer were performed. In 1980 and 1986 Bindoni et al. published papers showing that medial hypothalamic lesions led to a significant potentiation of Yoshida ascites tumor proliferation in rats, along with Ehrlich's tumor and L1210 ascites tumor proliferation in mice, and significantly increased cancer cell proliferation in inoculated ascites and solid tumors in mice and rats.^124^, ^125^ Also, pinealectomy has been associated with an increased incidence of experimentally induced breast tumors in rats, a phenomenon that was reversed by melatonin administration.^126^ Later, in 2008 Sarkar et al. demonstrated a marked protective effect on carcinogen‐induced prostate cancer in rats with implanted β‐endorphin‐synthesizing cells in the hypothalamus. The authors suggested that this effect was a consequence of potentiating the functions of innate immune components and reduction of inflammatory processes in prostate tissue.^127^ Thus, the above‐mentioned studies demonstrated the existence of a modulatory effect of certain brain structures on the initiation and progression of cancer. Most probably, this effect is mediated by those areas of the brain involved in important systemic homeostatic functions (e.g., hypothalamic nuclei).

### 1.7 Cancer affects the nervous system

The research of the neurobiology of cancer is focused mainly on investigating the modulatory effect of the brain or peripheral nerves on the initiation and progression of cancer. However, interactions between the nervous system and cancer are bidirectional. A more detailed study of the effects of cancer on the nervous system has only begun in the last two decades. Accumulated data indicate that cancer “manipulates” the host via modulation of host's nervous system activity. The goal of this manipulation is to further potentiate the proliferation of cancer cells and the development of metastases via modulating processes related to nervous system‐cancer interactions at the levels of the tumor micro‐ and macroenvironments.

#### 1.7.1 Cancer affects peripheral nerves

The effect of cancer on peripheral nerves responsible for induction of cancer pain is relatively well described. However, it has been found only recently that cancer cells produce nerve growth factor and therefore stimulate the ingrowth of new axons into the tumor tissue.^128^, ^129^ Because certain neurotransmitters (e.g., norepinephrine) released in the cancer microenvironment potentiate tumor growth, increased amount of cancer cells might further increases the production of nerve growth factor by cancer cells, and therefore positive feedback loop might be established, leading to potentiation of tumor growth.^130^

#### 1.7.2 Cancer affects the brain

Animal experiments performed in the last two decades have shown that besides induction of neoaxonogenesis, peripheral cancer affects several brain structures and circuits. Molecules released by cancer tissue affects many brain functions, including cognition,^131^ mood,^132^ sleep,^133^ metabolism,^134^ and visceral perception.^135^, ^136^

In 2005, Konsman and Blomqvist described changes in the activity of brain structures related to energy balance in tumor‐bearing animals. These authors have shown that the activity of forebrain structures in cachexic rats differed compared to control animals.^134^ Later, in 2013, Lackovicova et al. have shown that fibrocarcoma activated rat neurons in hindbrain structures and that this activation changed over time and differed between intraperitoneal and subcutaneous tumors.^135^ Recently, Horvathova et al. have shown that melanoma in mice affected brain structures related to visceral sensation, autonomic functions, cognition, and food intake.^136^

Several findings indicate that cancer also induces alteration of brain circuits participating in the regulation of energy balance and therefore these alterations might participate in the development of cancer‐induced anorexia and cachexia. Even if the research of cancer cachexia is traditionally focused preferentially on processes taking place in peripheral, metabolically active tissues (e.g., skeletal muscles, fat tissue, liver), the regulation of food intake and energy balance is orchestrated by the brain, particularly by the hypothalamus. Importantly, cytokines and other molecules released by the tumor micro‐and macroenvironment are able to induce inflammation in hypothalamic nuclei. It was also found that alteration of hypothalamic neuronal regulatory circuits caused by neuro‐inflammation might lead to the dysregulation of metabolism and alteration of energy balance and thus contribute to the development and progression of anorexia and cachexia in animal models of cancer and cancer patients.^137^, ^138^

Because cancer‐induced alteration of brain functions might further affect cancer growth and metastasis, therefore this factor might represent an additional hallmark of cancer (Figure 4). However, it is necessary to note that investigating the effect of cancer on the brain in human studies is difficult, because diagnosis of cancer itself induces high stress that affects brain functions. Therefore, it is difficult to distinguish between cancer‐related and stress‐related changes in the brain of oncological patients. For that reason, animal models of cancer are more appropriate for investigation of cancer's effects on the brain.

### 1.8 Complexity of the nervous system effects on cancer

Accumulated data have clearly shown that the nervous system affects both the cancer micro‐ and macroenvironments. At the level of the cancer microenvironment, in addition to its effect on DNA mutations and repair, oncogenic signaling, and tumor‐related immunity, the nervous system also affects other hallmarks of cancer as defined by Hanahan and Weinberg.^139^ It has been demonstrated that the nervous system affects the ability of cancer cells to sustain proliferative signaling, evade growth suppressors, resist cell death, enable replicative immortality, induce angiogenesis, activate invasion and metastasis, and reprogram energy metabolism.^130^ At the level of the cancer macroenvironment, the nervous system influences all organ systems which participate in the modulation of cancer growth and whose activity is affected by cancer. Bidirectional interactions between brain and gut microbiota might play a role in cancer, as well.^1^

### 1.9 Therapeutic and preventive implications of the neurobiology of cancer

**FIGURE 5:**
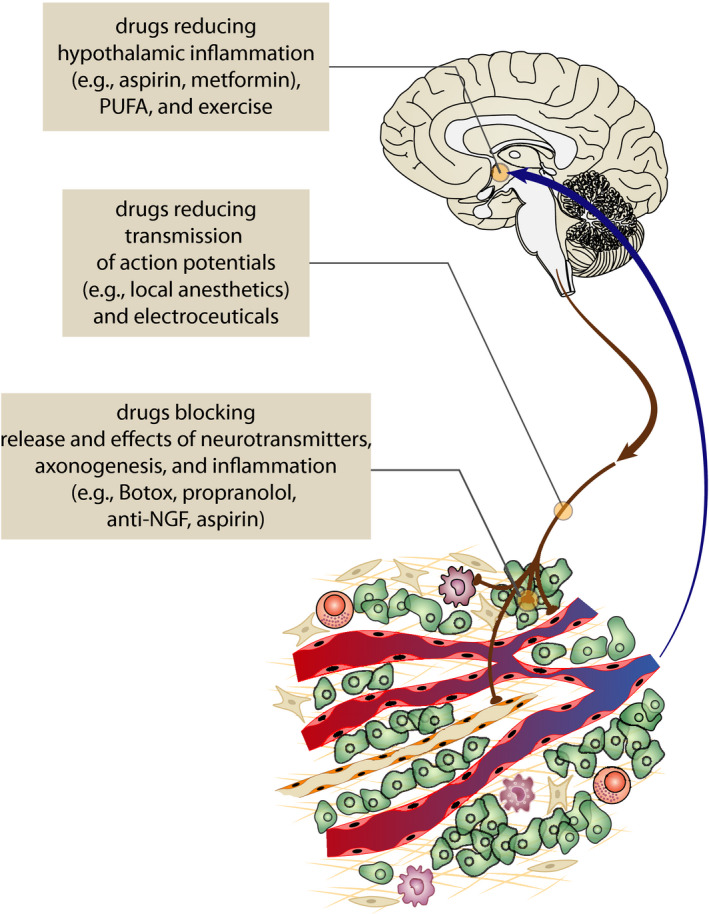
Research of the neurobiology of cancer has created a basis for the introduction of new treatment approaches in oncology. Repurposed and new drugs, as well as electroceuticals, might be used to reduce the stimulatory effect of the nervous system on cancer initiation and progression. In addition, suppression of the adverse effects of cancer on the brain might be useful for treatment of cancer anorexia and cachexia. NGF ‐ nerve growth factor; PUFA—polyunsaturated fatty acids

In the last years, research related to the neurobiology of cancer has rapidly expanded and the mechanisms and pathways mediating the influence of the nervous system on cancer development and progression as well as mechanisms mediating the effects of cancer on the nervous system have been elucidated in more detail. Based on these findings, several new approaches that can be utilized in the treatment and prevention of cancer have emerged (Figure 5).^140^ In addition, some practical questions raised, for example as whether it is appropriate to use nerve‐sparing approaches during recession of primary prostate cancer, even if it is not associated with an increased risk of relapse in short‐term and middle‐term follow‐up.^141^

#### 1.9.1 Treatment of cancer

New approaches in cancer treatment might include: (a) non‐pharmacological modalities reducing the impact of adverse psychosocial factors on the organism (e.g., psychotherapy, yoga, autogenic training, and biofeedback), (b) reduction of transmission of signals from nerves to cancer by surgical transection of these nerves or by repurposed drugs approved for non‐oncological indications (e.g., β‐blockers, Botox), (c) drugs reducing the ingrowth of new nerves into cancer tissue (e.g., antibodies against nerve growth factors), (d) modulation of electrical activity of nerves innervation cancer tissue (e.g., local anesthetics, electroceuticals), (e) reduction of the development of metastasis after resection of primary cancer tissue (e.g., propranolol and COX‐2 inhibitors^142^, ^143^), and (f) reduction of hypothalamic inflammation by peripherally and centrally acting anti‐inflammatory drugs (e.g., metformin, aspirin, polyunsaturated fatty acids) or by regular exercise.

#### 1.9.2 Augmentation of cancer treatment efficacy

Suppression of adverse effects of the nervous system on tumor micro‐ and macroenvironments might also be utilized for increasing the efficacy of conventional cancer treatment. For example, it was shown by the Repasky group that increased housing temperature attenuates the activity of the sympathoadrenal system and therefore increased the efficacy of checkpoint inhibitor immunotherapy and radiation therapy in animal models of cancer.^144^, ^145^, ^146^, ^147^, ^148^ The efficiency of cancer treatment might also increase β‐blockers and drugs reducing the effect of the norepinephrine co‐transmitter, neuropeptide Y. Recent studies have shown that propranolol reduced resistance and increased sensitivity to chemotherapy in patients with sarcoma or ovarian cancer^149^, ^150^ and inhibited cellular and molecular pathways associated with adverse outcomes in hematopoietic cell transplant recipients.^151^ Finally, it has been shown that nerves releasing neuropeptide Y might be involved in radiation therapy resistance.^152^

#### 1.9.3 Prevention of cancer development

Based on the recent findings of the neurobiology of cancer, some new preventive approaches that reduce activation of the sympathoadrenal system might also be recommended to cancer patients or individuals with increased genetic cancer risk or exposed to carcinogens. These approaches might include preventive treatment by β‐blockers to reduce the stimulatory effect of sympathoadrenal system on cancer development. In some cancers (e.g., gastric, prostate) reduction of parasympathetic nerve activity might also provide a beneficial effect.^80^, ^116^ Because cold stress activates brain structures regulating the activity of the sympathoadrenal system and nicotine activates sympathetic postganglionic neurons, reduction of cold effect on the organism (e.g., by warm clothes)^153^ and avoidance of combustible and electronic cigarettes containing nicotine^154^ might represent other preventive approaches useful in oncology.

## 2 CONCLUSION

A historical overview of observations, findings, and research on the neurobiology of cancer demonstrates that the role of the nervous system in cancer has been in the spotlight for centuries. Humoral theories of diseases and the experiences of clinicians strongly indicated that cancer is at least partially of psychosomatic nature. Since the 1920’s, advances in the surgical treatment of tumors, and later the introduction of radiotherapy and chemotherapy in oncology, led to a gradual decline of interest in elucidating the role of the nervous system in cancer. During this period, the previous psychosomatic view of tumors was replaced by the view of tumors as diseases limited to tumor tissue that can be surgically removed or treated with radiotherapy and chemotherapy. It was only later when a more important role began to be assigned to the nervous system, when in addition to the tumor microenvironment, researchers and oncologists began to refocus on the importance of the tumor macroenvironment.

Recently, combined neuroscientific and oncological research have created the basis for the neurobiology of cancer, which has brought a new, more complex view of the biology of cancer and elucidated the role that the nervous system plays in this disease. The neurobiology of cancer is uncovering new pathways and mechanisms participating in tumorigenesis, proliferation of cancer cells, and the development of metastasis. Based on these findings, another two hallmarks of cancer have emerged, specifically cancer tissue innervation and cancer‐induced changes in the nervous system. Importantly, the neurobiology of cancer also created a basis for repurposing of approved drugs as well as the introduction of new drugs abd methods that might be utilized for more efficient cancer treatment and prevention.

## CONFLICT OF INTEREST

I declare no competing interest.

## AUTHOR CONTRIBUTION

Manuscript and figures preparation.

## ETHICS STATEMENT

Not applicable.

## Data Availability

Not applicable.
